# Out‐of‐field doses from radiotherapy using photon beams: A comparative study for a pediatric renal treatment

**DOI:** 10.1002/acm2.13182

**Published:** 2021-02-05

**Authors:** Julie Colnot, Sofia Zefkili, Régine Gschwind, Christelle Huet

**Affiliations:** ^1^ Institut de Radioprotection et de Sûreté Nucléaire (IRSN) Service de Recherche en Dosimétrie Laboratoire de Dosimétrie des Rayonnements Ionisants Fontenay‐aux‐Roses France; ^2^ Institut Curie Service de Physique Médicale Paris France; ^3^ Université de Bourgogne‐Franche‐Comté LCE UMR 6249 Montbéliard France

**Keywords:** advanced radiotherapy, peripheral doses, radiochromic films, treatment planning system

## Abstract

**Purpose:**

First, this experimental study aims at comparing out‐of‐field doses delivered by three radiotherapy techniques (3DCRT, VMAT (two different accelerators), and tomotherapy) for a pediatric renal treatment. Secondly, the accuracy of treatment planning systems (TPS) for out‐of‐field calculation is evaluated.

**Methods:**

EBT3 films were positioned in pediatric phantoms (5 and 10 yr old). They were irradiated according to four plans: 3DCRT (Clinac 2100CS, Varian), VMAT (Clinac 2100CS and Halcyon, Varian), and tomotherapy for a same target volume. 3D dose determination was performed with an in‐house Matlab tool using linear interpolation of film measurements. 1D and 3D comparisons were made between techniques. Finally, measurements were compared to the Eclipse (Varian) and Tomotherapy (Accuray) TPS calculations.

**Results:**

Advanced radiotherapy techniques (VMATs and tomotherapy) deliver higher out‐of‐field doses compared to 3DCRT due to increased beam‐on time triggered by intensity modulation. Differences increase with distance to target and reach a factor of 3 between VMAT and 3DCRT. Besides, tomotherapy delivers lower doses than VMAT: although tomotherapy beam‐on time is higher than in VMAT, the additional shielding of the Hi‐Art system reduces out‐of‐field doses. The latest generation Halcyon system proves to deliver lower peripheral doses than conventional accelerators. Regarding TPS calculation, tomotherapy proves to be suitable for out‐of‐field dose determination up to 30 cm from field edge whereas Eclipse (AAA and AXB) largely underestimates those doses.

**Conclusion:**

This study shows that the high dose conformation allowed by advanced radiotherapy is done at the cost of higher peripheral doses. In the context of treatment‐related risk estimation, the consequence of this increase might be significative. Modern systems require adapted head shielding and a particular attention has to be taken regarding on‐board imaging dose. Finally, TPS advanced dose calculation algorithms do not certify dose accuracy beyond field edges, and thus, those doses are not suitable for risk assessment.

## 1 Introduction

Recently, early diagnosis and improvements in treatment techniques and therapeutic strategies have led to an increasing success of cancer treatments.^1^ Consequently, patients’ life expectancy following cancer is increasing and more patients will survive long after treatments. Among the different techniques involved, radiation therapy is nowadays used in more than 50% of cancer treatments^2^, ^3^ and its efficacy has been largely acknowledged. The most advanced techniques enable conformal dose distribution to the tumor volume reducing adjacent organs doses. This precision in dose delivery is carried out thanks to multiple beam incidences, beams’ intensity modulation, and a precise patient positioning using on‐board imaging systems. However, modern radiotherapy inevitably increases the volume of normal tissue exposed to ionizing radiation from treatment beams themselves, from out‐of‐field radiation,^4^ and also from daily imaging.^5^

The exposure of normal tissues may lead to adverse effects following treatments. Although deterministic effects only appear at high doses, stochastic effects such as second cancers or cardiac diseases can be related to low doses exposure and can occur years after treatment. As life expectancy after cancer is increasing and modern techniques are now widely used, late treatment‐related side effects are becoming an important concern. Besides, this issue is even more important for young patients because of their high organs radiosensitivity and their long‐term survival following primary cancer.^6^ The risks related to medical exposure are determined from epidemiological studies which aim at correlating doses to observed side effects. Thus, a precise knowledge of the dose distribution delivered to patients and to healthy organs is needed to enhance the prediction of adverse effects risks and their reduction. Modern treatment planning systems (TPS) enable a precise determination of the doses delivered within the treatment beams but for locations outside the treatment field edge, some tend to largely underestimate the doses ^7^. In addition, the accuracy of out‐of‐field dose determination of some recent TPS algorithms is not well known. Therefore, they cannot be used to estimate related adverse effects risks. Furthermore, there is few experimental data regarding out‐of‐field doses acquired in realistic conditions (anthropomorphic phantoms), in particular for recent radiotherapy techniques and for pediatric patients.

The Institut Curie (Paris, France) has a long experience in pediatric oncology. In this institute, photon radiotherapy is particularly involved in the treatment of pediatric abdomino‐pelvic cancers and among them, our work focuses on renal tumors which are frequently represented. Before the generalization of intensity modulation, the standard radiotherapy modality used to treat those cancers was conventional three‐dimensional conformal treatment (3DCRT) consisting in two anteroposterior beams. Now, they are typically treated using volumetric arc therapy (VMAT) using conventional linear accelerators and sometimes using tomotherapy units.

In this context, the first aim of this experimental study is to compare the peripheral doses delivered by two advanced techniques (VMAT and tomotherapy) and by conventional 3DCRT for pediatric abdomino‐pelvic cancers treatment. In this study, in addition to the Clinac 2100, the latest generation accelerator Halcyon version 2.0 (Varian Medical System), newly installed at the Institut Curie was also included to deliver VMAT plans as its use may be considered in the future for pediatric patients. This accelerator is a single‐beam 6 MV‐FFF system equipped with dual‐layer multi‐leaf collimator (MLC) and fixed primary and secondary collimators.^8^, ^9^ Clinically relevant treatment plans according to commonly used planning protocols were prepared for two anthropomorphic pediatric phantoms (5 and 10 yr old). Peripheral dose comparison was performed thanks to EBT3 film measurements placed in these phantoms in order to overcome the possible inaccuracy of TPS algorithms outside the treatment field.^7^ The measured out‐of‐field doses were reconstructed in 3D by linear interpolation using original in‐house Matlab scripts.^10^ The second aim of the present study is to evaluate the accuracy of two TPS for out‐of‐field dose calculation. For that purpose, TPS calculated doses obtained for the two phantoms and the different techniques are compared to the experimental doses obtained from the film measurements. The TPS Eclipse™ (Varian MS) and tomotherapy (Accuray Inc., Sunnyvale, CA, USA) were studied.

## 2 Materials and Methods

### 2.A Treatment planning

**FIG. 1:**
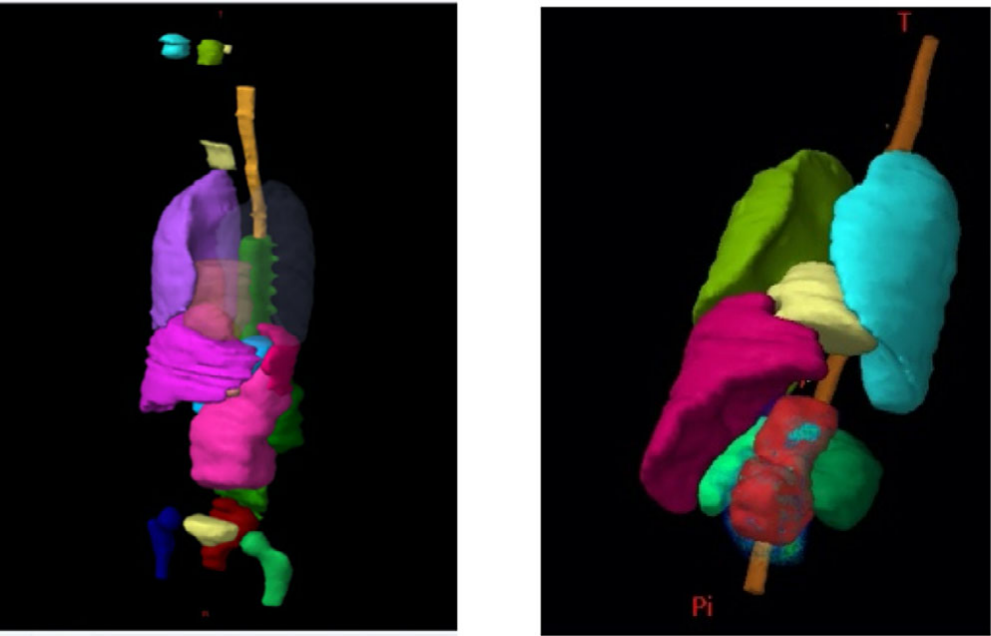
Organs outlined inside the 5‐year‐old (left) and 10‐year‐old (right) anthropomorphic phantom using the Eclipse™ TPS. PTVs are in pink and red, respectively.

The heterogeneous ATOM^®^ dummies (CIRS, Norfolk, VA) representing children aged 5 and 10 yr old were used in this study. They were scanned with a 3 mm slice thickness using an Aquilion LB (Toshiba) CT scanner at the Institut Curie (Paris, France) according to a clinical protocol. Two pediatric patients, morphologically similar to the two phantoms and previously treated at the Institut Curie for renal tumors, were then selected. Those patients were extracted from a pediatric patient cohort treated for renal tumors gathered by a radiotherapist. Different parameters were analyzed in that cohort: patient age, patient size, patient weight, PTV volume, and PTV localization. The two selected patients corresponded to the median case for each age group. Using the Eclipse™ TPS (Varian Medical System), the patients’ CT images and their outlined structures were registered on the phantoms’ CT images using deformable registration. In order to obtain realistic planning treatment volumes (PTV) and organs shapes in the phantoms, the registered structures were copied on phantoms CT images and manually processed to avoid any overlap. The PTV including the clinical target volume (CTV) with a 5 mm margin and the vertebrae in its immediate proximity were of 726 cc and of 382 cc for the 5‐year‐old and 10‐year‐old phantoms, respectively. For the 10‐year‐old phantom, the PTV is central and located slightly to the left of the lumbar rachis. For the 5‐year‐old phantom, the target extends in front of the left kidney (Fig. 1). The structures obtained from the patients’ files are the liver, the kidneys, the spinal cord near the CTV, the digestive system, and the vertebrae. As for clinical practice, only a small portion of the patient body is scanned, additional organs were manually outlined within the phantoms such as eyes, thyroid, lungs, heart, bladder, and rectum (Fig. 1). The RVR (remaining volume at risk), defined by the difference between the volume of the body contour and that of the CTV and the outlined organs, was also studied in this work.

For each phantom, four treatment plans were developed and optimized in accordance with clinical constraints and the institute pediatric experience. Table 1 lists the parameters of the four treatment plans. The VMAT and 3DCRT plans were optimized using the AAA algorithm (Eclipse™, Varian) and then recalculated using Acuros^®^ (except for the Halcyon plan) in dose to water by keeping the same MU per beam. Tomotherapy plans were calculated with the dedicated Tomotherapy TPS (Accuray). The dose calculation grid includes the entire body of phantoms. To study the two jaw modes available on the tomotherapy system (static and dynamic), the static mode was used for the 5‐year‐old plan whereas the dynamic delivery was used for the 10‐year‐old plan. The jaw width was set to 2.5 cm for the two phantoms. All the plans were generated with 6 MV beams, the tomotherapy and Halcyon units use FFF beams. 21 Gy (14 fractions) were prescribed to the PTV and the dose was normalized so that the mean PTV dose matches the prescribed dose. In the following, the VMAT plan performed with the Clinac 2100CS accelerator is referred as VMAT plan whereas the one performed with the Halcyon system is referred as Halcyon plan.

**TABLE 1:** Treatment plans prepared in this study for the two phantoms and for the four radiotherapy techniques.

Radiotherapy techniques	Treatment delivery	Linear accelerator	Calculation algorithm	Phantom	
				5‐year‐old	10‐year‐old
				Total MU/beam‐on time per fraction	Total MU/beam‐on time per fraction
3DCRT	Two anteroposterior beams	Clinac 2100CS (Varian)	AAA v13.6.23 Acuros^®^ v13.6.23	2324	2520
VMAT	Two lateral half‐arcs	Clinac 2100CS (Varian)	AAA v13.6.23 Acuros^®^ v13.6.23	6132	4746
VMAT	Two lateral half‐arcs	Halcyon v2.0 (Varian)	Halcyon v15.6.03	5782/30 s	6132/30 s
Tomotherapy	Helical	Tomotherapy Hi‐Art (Accuray)	Accuray Precision v1.1.1.1	3460/4 min	3642/4.25 min

### 2.B Radiochromic film dosimetry and phantom irradiation

For dose measurements on phantoms, EBT3 radiochromic films (Ashland) were used. EBT3 films were chosen for out‐of‐field dose measurements as their response proves to have little dependence with energy.^12^, ^13^, ^14^, ^15^, ^16^ Moreover, they allow 2D dose measurements and they can easily be housed in anthropomorphic phantoms. Measurements were performed according to a rigorous protocol (cutting, calibration, readout) developed in our laboratory.^11^ In particular, it is based on a pixel‐to‐pixel background subtraction method with the use of the red channel only in order to overcome the limits of the multichannel correction method at low doses.^15^ Measurements uncertainty was assessed as described in.^11^ This protocol leads to dose measurements with a standard deviation of 2.9% (1 sigma) in the 0.5–4.0 Gy dose range (reaching 4.5% (1 sigma) for doses below 0.5 Gy). Besides, this protocol was confronted to a Farmer ion chamber for off‐axis dose measurements in a previous work^17^ and demonstrates good agreement with maximum discrepancies of 20% up to 6 cGy.

**FIG. 2:**
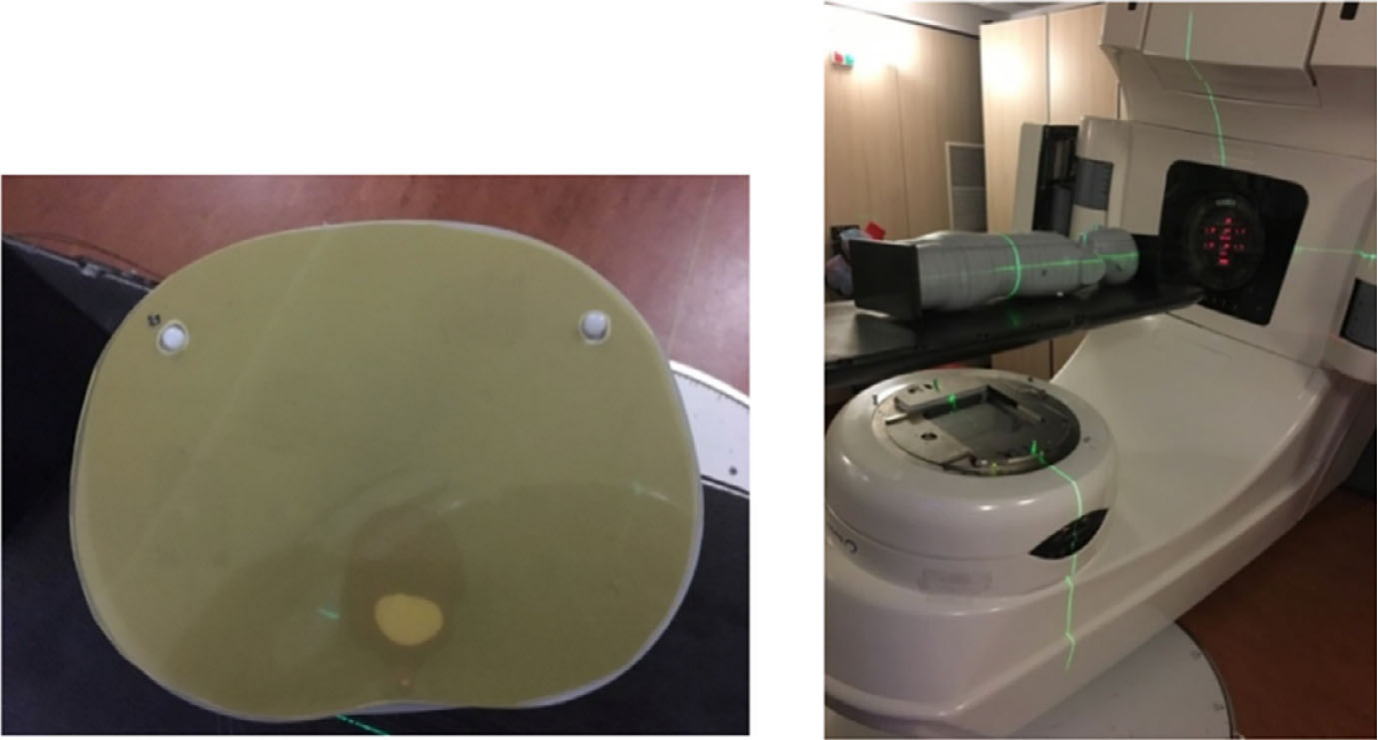
Radiochromic film placed between slices inside the ATOM dummy (left) and dummy positioning for the 3DCRT treatment (right).

The calibration was made at the Institut Curie with a Clinac 2100CS linear accelerator (Varian MS); calibration films were irradiated the same day as the phantoms. The films were calibrated from approximately 1.3 cGy (2 MU) to 24 Gy using 18 dose points (2 films per dose) between tissue‐equivalent slabs at 10 cm in depth with a 10 × 10 cm^2^ field (SSD = 100 cm). Besides, two overlapping fit curves (from 0.013 Gy to 5 Gy and from 3 Gy to 24 Gy) have been used to make sure that low doses are perfectly represented by the final calibration curve. For the phantoms’ measurements, 31 films and 25 films were cut to fit between the slices of the 10‐year‐old and 5‐year‐old phantoms, respectively (Fig. 2 left). Those measurements were performed for each radiotherapy technique.

The phantoms were filled with the EBT3 films and irradiations were performed according to the prepared treatment plans at the Institut Curie (Fig. 2 right). For each plan, the 14 fractions were delivered successively without any imaging between them. Thus, no doses from on‐board imaging were delivered to the films. The irradiations lasted one hour and a half, one hour, and less than half an hour for the tomotherapy, VMAT with Clinac 2100CS, and the 3DCRT and Halcyon plans, respectively.

### 2.C Data analysis

Data and films analyses were carried out using in‐house Matlab scripts (Matlab R2013b and Image Processing Toolbox, The MathWorks, Inc.).^10^ The different steps of the analysis are described as follows:

1. Conversion of films optical density into absorbed dose to water,
2. Import of DICOM files (CT images, RD, RS) in Matlab and data formatting,
3. Spatial registration of the measured dose distribution with phantom CT images,
4. Dose reconstruction in 3D by linear interpolation between the slices,
5. Data analysis for radiotherapy techniques comparison and TPS evaluation:
  ‐ 1D: dose profiles in the craniocaudal axis perpendicular to films orientation; each point of those profiles is obtained by averaging dose over 9 voxels (voxels of 1 mm × 1 mm × 1 mm),
  ‐ 2D: organs dose difference for the TPS evaluation only. The 2D mean organ doses are calculated by averaging the doses measured with films intersecting outlined structures. Similarly, computed 2D mean doses are obtained by averaging the doses obtained with the TPS at the intersection between films’ location and outlined structures. This analysis is done for every contoured structure intersecting films: it includes organs located inside treatment beams.
  ‐ 3D outside the treatment field border (below 5% of the prescribed dose): mean organ doses and DVH obtained from doses reconstructed in 3D and calculated doses. For organs partially within the treatment beams, only the volume located outside the beams was considered for this dose determination.

The comparison of out‐of‐field doses delivered by the different radiotherapy techniques is based on the measured doses obtained from the films. This experimental comparison enables a robust comparison as it overcomes the potential TPS inaccuracy of dose calculation at distance from PTV. In the second part, the accuracy of TPS dose calculation is studied by comparing measured and calculated dose.

## 3 Results

### 3.A Comparative study of out‐of‐field doses delivered by radiotherapy techniques

#### 3.A.1 Five‐year‐old phantom

##### 1‐D comparison

**FIG. 3:**
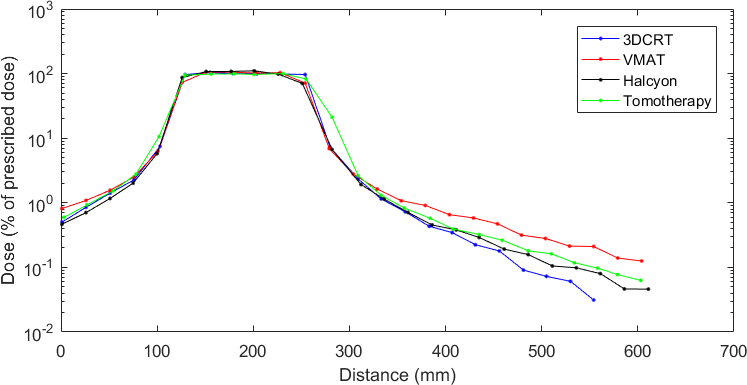
Measured dose profiles (% of prescribed dose) in the feet–head axis for the 5‐year‐old phantom.

Figure 3 shows dose profiles obtained from the films’ measurements in a feet–head axis perpendicular to the films for the four radiotherapy techniques: 3DCRT, VMAT using the Clinac 2100CS accelerator and Halcyon, and tomotherapy. Those profiles are positioned within the phantom water‐equivalent material. The last two points located at the top of the head in 3DCRT are inferior to 2 mGy and were not represented here. Away from PTV, the dose decreases up to approximatively 2 cGy with modern techniques.

As expected, in the PTV high dose region, the four radiotherapy techniques deliver similar dose levels due to identical prescription and dose normalization. Indeed, mean differences against 3DCRT doses are of 1.20% (σ = 1.03%), −3.86% (σ = 10.4%), and −2.52% (σ = 14.2%) for tomotherapy, VMAT, and Halcyon, respectively. Close to the treatment beams, tomotherapy delivers the highest doses: the discrepancy reaches 218% at 2.5 cm from PTV in the head direction. The other techniques show similar dose levels at this location. Away from PTV, that is, beyond approximatively 2.5 cm from field edge, the delivered doses decrease with distance and the discrepancies between techniques increase. 3DCRT better spares normal tissues in comparison with modern radiotherapy techniques and the highest doses were obtained with VMAT. The mean differences on the entire profiles in comparison with 3DCRT are of 53.9% (σ = 85.6%), 93.3% (σ = 148%), and 16.4% (σ = 108%) for tomotherapy, VMAT, and Halcyon, respectively. Moreover, the largest discrepancies reach 350%, 592%, and 424% at 30 cm from field edge for tomotherapy, VMAT, and Halcyon, respectively.

##### 3D doses delivered outside the treatment field border

Mean doses reconstructed in 3D outside treatment beams are given in Table 2 in percent of the prescribed dose for the different radiotherapy techniques. They range from 0.04% of the prescribed dose (pituitary and eye) to 2.73% of the prescribed dose (rectum). The least exposed organs are located in the head with doses between 1 cGy (0.04% of prescribed dose) and 4 cGy (0.20% of prescribed dose) depending on technique. The dose differences compared to 3DCRT are listed in Table 3.

**TABLE 2:** Mean doses (% of prescribed dose) reconstructed in 3D outside the treatment beams for the 5‐year‐old phantom.

	Heart	Spinal cord	Lung R	Lung L	Thyroid	Rectum	Bladder	Eye R	Eye L	Pituitary	H & N	Pelvis	RVR
3DCRT	1.51	0.50	0.78	1.11	0.19	2.15	2.54	0.04	0.06	0.04	0.07	1.07	0.54
VMAT	1.89	0.83	1.42	1.36	0.49	2.20	2.48	0.21	0.23	0.20	0.23	1.38	0.81
Halcyon	1.44	0.52	1.05	0.92	0.25	1.91	2.07	0.09	0.09	0.09	0.10	1.03	0.54
Tomotherapy	1.76	0.61	1.11	1.22	0.29	2.45	2.73	0.12	0.12	0.12	0.14	1.31	0.67

**TABLE 3:** Relative 3D dose difference between VMAT, tomotherapy, Halcyon and 3DCRT for the 5‐year‐old phantom.

	VMAT/3DCRT	Tomotherapy/3DCRT	Halcyon/3DCRT
Heart	25.4	16.9	−4.56
Spinal cord	66.6	22.7	3.44
Lung R	82.2	41.9	35.0
Lung L	21.9	9.99	−17.0
Thyroid	153	50.2	28.5
Rectum	1.95	13.9	−11.2
Bladder	−2.44	7.22	−18.8
Eye R	365	175	101
Eye L	306	115	63.0
Pituitary	370	190	125
Head and Neck	234	108	44.4
Pelvis	29.3	23.1	−3.50
RVR	48.3	24.1	0.29

Given the results presented in Tables 2 and 3, 3DCRT, and Halcyon enable a better sparing of organs located outside the treatment beams in the head direction in comparison with the other techniques. This result is consistent with previous 1D results. In the head direction, 3DCRT better spares all the organs in comparison with VMAT and tomotherapy and dose levels are similar to Halcyon (for the heart for instance). The most important relative discrepancies are obtained for head and neck organs located far from the PTV (Table 3), reaching 370% for pituitary in VMAT.

Doses delivered outside treatment beams are globally lower with tomotherapy than with VMAT. Thus, for organs more distant from PTV, VMAT delivers the higher dose levels: the differences with tomotherapy reach 44.1% for the eyes and 16.3% for the out‐of‐field RVR.

The relative difference between VMAT, tomotherapy, and 3DCRT is also important for the pelvic region located closer to the PTV (Table 3). Regarding modern techniques, the measured 3D doses are higher with tomotherapy than with VMAT for the pelvic organs close to the PTV (rectum, bladder) as observed in 1D. The dose discrepancies reach 11.7% and 9.90% for rectum and bladder, respectively. However, when the entire pelvic region is considered, the mean dose delivered in VMAT is superior of 4.84% in comparison with tomotherapy. Regarding Halcyon, the dose delivered to the entire pelvis is similar to 3DCRT (Table 3) but this technique largely enables to spare rectum (−11.2%) and bladder (−18.8%) compared to 3DCRT.

#### 3.A.2 Ten‐year‐old phantom

##### 1‐D comparison

**FIG. 4:**
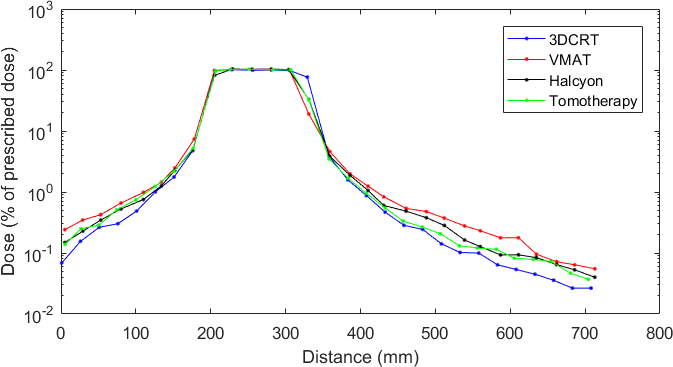
Measured dose profiles (% of prescribed dose) in the feet–head axis for the 10‐year‐old phantom.

Figure 4 shows the measured dose profiles in the feet–head direction perpendicular to the films for the four radiotherapy modalities. The results are consistent with those obtained for the 5‐year‐old phantom. On those profiles, the lowest doses are approximatively of 4 mGy.

As expected, in the high dose region, the four techniques deliver similar dose levels with mean differences against 3DCRT of 2.08% (σ = 1.19%), of 1.48% (σ = 2.07%), and of −0.251% (σ = 8.95%) for VMAT, tomotherapy, and Halcyon, respectively. Contrary to the 5‐year‐old phantom, close to the PTV in the caudal direction, the four techniques deliver similar doses whereas in the cranial direction, the dose levels are 75.2% higher in 3DCRT than in VMAT. Moreover, in the cranial direction, the doses measured are similar in tomotherapy and 3DCRT from 5 cm to 17.5 cm from field edge.

##### 3D doses delivered outside the treatment field border

Table 4 reports the mean doses reconstructed in 3D expressed in percent of the prescribed dose for organs located outside beams in the 10‐year‐old phantom. They range from 0.02% of the prescribed dose (pituitary and eye) to 1.70% of the prescribed dose (heart). The lowest dose levels are obtained for the head and neck organs (4 mGy for pituitary). Table 5 gives relative dose differences in comparison with 3DCRT.

**TABLE 4:** Mean doses (% of prescribed dose) reconstructed in 3D outside the treatment beams for the 10‐year‐old phantom.

	Heart	Spinal Cord	Lung R	Lung L	Digestive	Thyroid	Ovary R	Ovary L	Uterus	Bladder	H & N	Pelvis	Eye R	Eye L	Pituitary	Rectum	RVR
3DCRT	1.39	0.35	0.50	0.57	0.72	0.06	0.56	0.56	0.32	0.38	0.03	0.33	0.04	0.04	0.02	0.35	0.305
VMAT	1.70	0.50	0.94	0.89	1.05	0.27	0.95	0.86	0.58	0.75	0.09	0.66	0.11	0.09	0.06	0.60	0.742
Halcyon	1.51	0.37	0.79	0.66	0.83	0.15	0.80	0.65	0.47	0.58	0.07	0.43	0.06	0.06	0.05	0.47	0.525
Tomotherapy	1.15	0.31	0.57	0.60	1.06	0.11	1.15	1.11	0.61	0.60	0.05	0.46	0.03	0.04	0.07	0.63	0.366

**TABLE 5:** Relative difference (%) between 3D interpolated doses for the 10‐year‐old phantom.

	VMAT/3DCRT	Tomotherapy/3DCRT	Halcyon/3DCRT
Heart	22.1	−17.6	8.28
Spinal cord	41.2	−12.2	3.86
Lung R	87.1	14.1	57.1
Lung L	57.5	6.26	16.8
Digestive	45.9	46.6	15.2
Thyroid	339	73.3	151
Ovary R	71.7	107	44.0
Ovary L	53.2	99.0	15.9
Uterus	80.9	90.3	45.9
Bladder	98.1	60.1	54.2
Head and neck	208	79.3	134
Pelvis	98.3	39.3	30.2
Eye R	167	−28.8	56.7
Eye L	134	1.66	56.9
Pituitary	234	287	191
Rectum	70.4	78.6	32.9
RVR	143	19.9	72.0

The results obtained are similar to the previous 1D analysis. Lower dose levels are globally obtained in 3DCRT in comparison with modern techniques away from beam edges and in particular against VMAT. Tomotherapy and Halcyon are the modern techniques that lead to the lowest peripheral doses. The Halcyon system enables lower out‐of‐field doses than VMAT for all organs and even lower doses to the pelvis compared to tomotherapy (uterus, ovaries, bladder, rectum). The largest discrepancies against 3DCRT are obtained for head and neck organs (Table 5). Regarding the upper body, the doses delivered to organs located partially within the beams or in their proximity (heart, lungs) are higher with VMAT than with tomotherapy.

### 3.B Performance of TPS algorithms

In this part, only the results obtained with the 5‐year‐old phantom are presented as results are identical between the two phantoms.

#### 3.B.1 Eclipse™ TPS

##### 1D comparisons

**FIG. 5:**
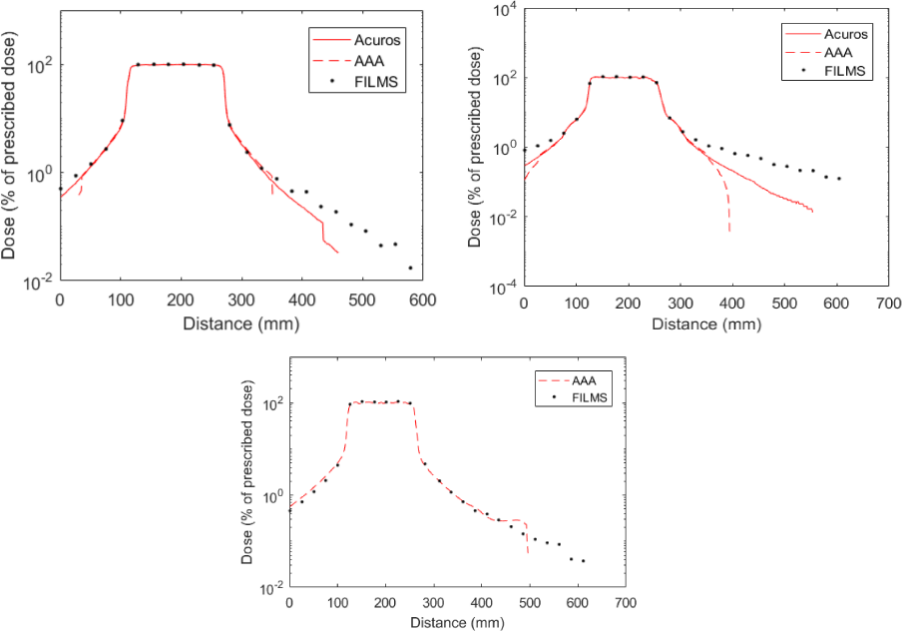
Dose profiles (% of prescribed dose) calculated with the Eclipse™ TPS and compared to radiochromic film measurements for the 5‐year‐old phantom; left: 3DCRT, right: VMAT; bottom: Halcyon.

Figure 5 shows the dose profiles calculated with the AAA and Acuros^®^ algorithms of the Eclipse™ TPS for 3DCRT, VMAT, and Varian Halcyon (only AAA) treatments. They are oriented in the feet–head direction within the 5‐year‐old phantom center. Even though the dose calculation was performed in the entire CT volume, the Eclipse™ TPS calculates positive doses only in a certain volume around PTV.

VMAT doses are calculated on a larger volume than for 3DCRT plans, in particular with the Acuros^®^ algorithm. Moreover, Acuros^®^ provides a larger dose evaluation than AAA for the two radiotherapy techniques. In our study, AAA calculates doses up to 10 cm from beam edge in 3DCRT and VMAT and up to approximatively 20 cm for the Halcyon system whereas Acuros^®^ calculates up to 15 cm in 3DCRT and up to 30 cm in VMAT. At the edge of PTV (up to approximatively 5 cm), a good agreement is obtained between measurements and the two algorithms in 3DCRT and with AAA using the Halcyon accelerator, but an underestimation is observed in VMAT reaching 40% at 5 cm from field edge.

At larger distance from PTV, Acuros^®^ and AAA underestimate delivered doses compared to measurements in 3DCRT and VMAT. This underestimation increases with distance to beams and is more important in VMAT as doses are calculated further. In VMAT, the doses calculated with AAA decrease rapidly at 10 cm from field edge whereas Acuros^®^ calculates a slower slope. Differences between Acuros^®^ and measurements reach −91.2% in VMAT and −60.4% in 3DCRT at 15 cm from PTV edge. Finally, AAA and measurements are in agreement for the Halcyon study, except from approximatively 20 cm of field edge where the TPS does not respect the dose decrease with distance and provides null values beyond that point. Regarding the AAA algorithm, the agreement between calculation and measurements proves to be different depending on radiotherapy technique (Fig. 5).

##### Organs doses

**FIG. 6:**
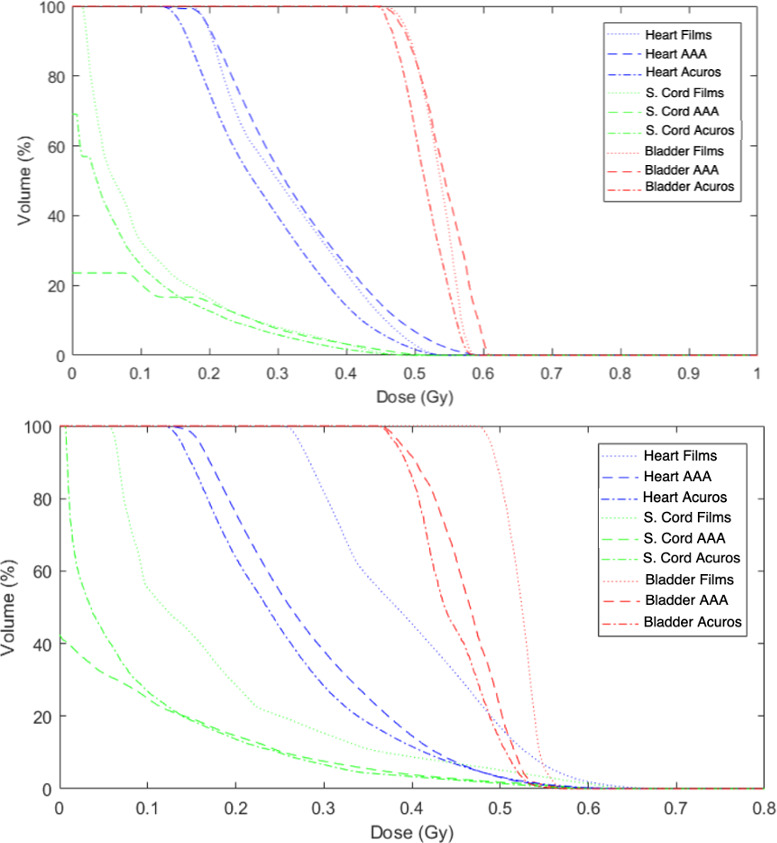
DVH obtained with AAA, Acuros^®^, and films measurements for three selected organs within the 5‐year‐old phantom for 3DCRT (top) and VMAT (bottom).

Mean dose differences obtained in 2D at films levels and in 3D using the reconstruction tool for organs located outside the beams are given in Table 6. Thus, the outlined organs which are not crossed by films are consequently not listed in this table. Moreover, DVH obtained with measurements and TPS are represented in Fig. 6 for 3DCRT and VMAT.

**TABLE 6:** Relative dose differences (%) between calculated and measured doses within organs in 2D and 3D.

Organs	3DCRT				VMAT				Halcyon	
	2D		3D		2D		3D		2D	3D
	AAA	Acuros^®^	AAA	Acuros^®^	AAA	Acuros^®^	AAA	Acuros^®^	AAA	AAA
Right eye	−100	−100	−100	−100	−100	−100	−100	−100	−100	−100
Left eye	−100	−100	−100	−100	−100	−100	−100	−100	−100	−100
Thyroid	−100	−85.1	−100	−77.1	−100	−84.1	−100	−82.2	46.7	25.0
Head and neck	−100	−88.0	−100	−94.0	−100	−89.2	−100	−89.6	−29.2	−39.4
Right lung	−4.32	−11.6	−16.7	−18.1	−18.2	−27.2	−29.9	−38.8	2.05	31.8
Left lung	−5.26	−6.04	−28.9	−17.9	−25.4	−28.3	−44.2	−46.6	−33.3	21.6
Heart	8.68	−4.24	3.94	−10.6	−13.1	−16.9	−28.4	−38.6	−33.0	−0.328
Spinal cord	−2.97	−3.87	−44.2	−27.6	−3.64	−4.43	−58.8	−50.8	−8.91	1.23
PTV	−0.09	−2.37	–	–	−1.30	−1.60	–	–	−4.82	–
Right kidney	−8.14	−5.06	–	–	−4.93	−6.82	–	–	−1.60	–
Left kidney	−0.345	−1.03	–	–	3.15	0.154	–	–	−4.84	–
Liver	−0.613	−0.575	–	–	−3.56	−3.48	–	–	−7.67	–
Stomach	−0.257	0.320	–	–	−0.676	1.19	–	–	2.52	–
Duodenum	−0.132	−0.236	–	–	−2.71	−1.12	–	–	−4.79	–
Spleen	1.83	1.45	–	–	2.70	2.12	–	–	−9.32	–
Pancreas	0.967	1.27	–	–	−2.36	−1.78	–	–	−4.61	–
Rectum	−2.14	−7.82	−2.42	−7.41	0.467	−2.84	−15.7	−18.9	−13.5	21.6
Bladder	4.52	−1.02	1.67	−3.54	−5.56	−8.14	−11.0	−14.4	24.4	23.7
Pelvis	−21.7	−12.8	−31.0	−17.8	−36.0	−34.1	−43.3	−39.5	20.3	18.7
RVR	−2.16	−2.61	−41.0	−23.2	−3.32	−3.53	−55.3	−49.8	−5.44	10.1

A good agreement is obtained between TPS and measurements for organs located totally or partially within the beams such as kidneys, duodenum, digestive apparatus, liver, pancreas, spleen, and stomach (dose difference below 9.32%). For spinal cord and RVR, the dose range is large as a portion of their volume is irradiated by treatment beams and other parts are far from PTV where the TPS gives only null values. The good agreement of mean doses between films and calculation in 2D does not allow to demonstrate the TPS dose calculation errors. DVHs of Fig. 6 highlights that TPS dose calculations are unsatisfying for organs partially irradiated by treatment beams such as the spinal cord for instance. Besides, in Table 6, larger discrepancies can be observed in 3D as only the organs volume located outside the beams is considered.

Discrepancies between TPS and measurements increase with distance to PTV as dose decrease. Those results are in agreement with dose profiles. Away from PTV, the two TPS algorithms underestimate delivered doses in comparison with measurements. This underestimation is particularly high for the furthest organs, that is, lungs and pelvic organs. Moreover, for most organs (eyes, thyroid, head, and neck region), TPS algorithms, in particular AAA, only give null values as previously shown. Thus, for remote organs, the Eclipse™ TPS cannot be used to estimate delivered doses: even though Acuros^®^ estimates dose further from PTV than AAA, it underestimates head and neck doses up to 50% and triggers important errors in DVH for organs partially in treatment beams (spinal cord for instance).

#### 3.B.2 Tomotherapy TPS

##### 1D comparison

**FIG. 7:**
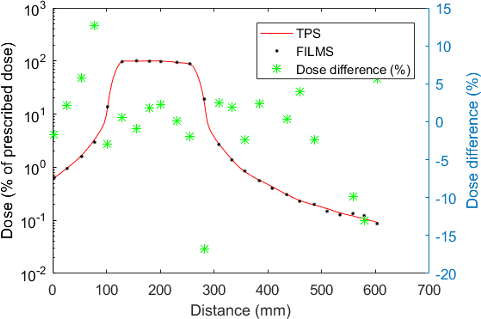
Dose profiles (% of prescribed dose) calculated with the Tomotherapy TPS and measured with films in the 5‐year‐old phantom and relative dose differences.

Figure 7 presents dose profiles measured with films and calculated using the Tomotherapy TPS in the center of the 5‐year‐old phantom within the water‐equivalent material. This graph is oriented in the feet–head direction.

Contrary to the Eclipse™ TPS, the Tomotherapy TPS (Accuray) calculates doses in the whole phantom volume, that is, up to 35 cm from PTV edge in this case. A good global agreement is obtained between planned and measured doses. The mean dose difference on the profile is of 0.73% (σ = 6.82%). The most important discrepancies are reached at the head level: a slight underestimation of 13.1% is observed for the TPS.

##### Organs doses

**FIG. 8:**
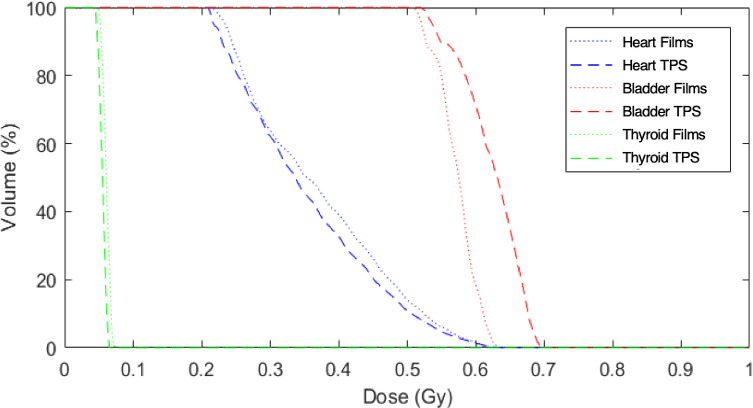
DVH obtained with the Tomotherapy TPS and measurements within the 5‐year‐old phantom.

Table 7 summarizes dose discrepancies between TPS and measurements for the different organs delineated in the phantom with in 2D at the film levels and in 3D outside the beams. Finally, Figure 8 represents DVHs obtained with the TPS and the 3D reconstruction tool.

**TABLE 7:** Dose differences between Tomotherapy TPS and film measurements (at films level and reconstructed in 3D outside the beams).

Organs	Dose differences (%)	
	2D	3D
Thyroid	−11.0	−9.71
Right eye	−36.4	−40.5
Left eye	−31.2	−33.6
Head and neck	−14.0	−13.4
Heart	−10.6	−4.04
Spinal cord	−2.99	2.03
Right lung	−7.52	−6.46
Left lung	−12.2	−10.0
PTV	−0.618	–
Liver	−3.70	–
Right kidney	−1.84	–
Left kidney	−0.710	–
Stomach	0.220	–
Duodenum	0.0779	–
Pancreas	1.57	–
Spleen	−1.48	–
Bladder	10.8	8.93
Rectum	−4.44	4.64
Pelvis	4.04	−0.37
RVR	−1.73	−3.87

A good global agreement is shown between calculated and measured doses in terms of mean doses and DVHs. Indeed, except for the head and neck region, discrepancies do not exceed 11% (as observed for the bladder in Fig. 8). A good agreement is obtained for organs remote from PTV such as the rectum. The most important differences are obtained for the eyes even though the discrepancy for the head and neck region is of 14%.

## 4 Discussion

### 4.A Comparative study of out‐of‐field doses delivered by radiotherapy techniques

Despite the difference in PTV size and morphology between the two phantoms, the results in terms of doses delivered outside treatment beams are consistent. For both phantoms, the highest doses measured at distance from PTV were obtained with modern techniques. The major difference between the two phantoms is that more organs are spared with 3DCRT than with modern techniques for the 10‐year‐old phantom. This is particularly observed for thoracic and abdominal organs such as digestive system and lungs. In fact, for this phantom, the organs are located farther from the high dose region than for the 5‐year‐old due to PTV size and phantom morphology. Moreover, the measured dose levels are lower with the 10‐year‐old phantom. Similarly, for this phantom, the relative discrepancies between the doses delivered with modern techniques and 3DCRT are greater.

The increase in dose outside the treated volume with modern radiotherapy can be explained by the longer beam‐on time of those treatments. Indeed, MU delivered in VMAT are 88% and 164% higher than in 3DCRT for the 10‐year‐old and 5‐year‐old phantoms, respectively. For tomotherapy, MU are not representative of the irradiation time as beam‐on time is superior of a factor of 2 in comparison with VMAT. The Halcyon system, using FFF beams, is the most effective modern modality with only 30 s of beam‐on time per fraction. The longer beam‐on time increases the scattered radiation and head leakage generated by the treatment. This leads to a larger patient exposure to secondary radiation which particularly contributes away from the target volume. In this case, as the same accelerator was used for VMAT and 3DCRT, the higher dose levels observed with VMAT are directly related to the increase in irradiation time. While comparing VMAT treatments, doses delivered by Halcyon to thyroid or head and neck region are reduced by a factor of 2 compared to the doses delivered with the Clinac 2100CS accelerator. These lower dose levels are also due to lower beam‐on time and reduced stray radiation induced by FFF beams in comparison with the FF beams of conventional accelerators. This larger exposure of healthy tissues induced by intensity‐modulated radiotherapy due to MU and beam‐on time increase was also shown by Wang and Xu.^18^ They also demonstrate the more beams are used in IMRT, the more peripheral doses are delivered to patients. Moreover, Stathakis *et al*.^19^ report that the whole‐body dose is increased by a factor 3 with IMRT in comparison to 3DCRT for a prostate treatment. They also highlight that this factor depends on beam energy, field size, and collimator rotation which affects leakage through this piece.

**FIG. 9:**
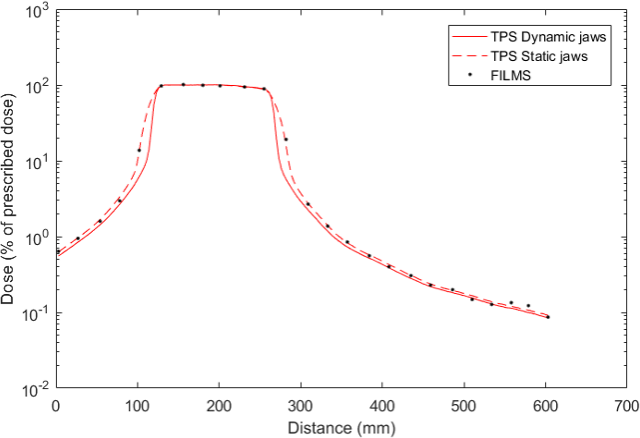
Dose profiles calculated by the Tomotherapy TPS with static and dynamic jaws in the 5‐year‐old phantom.

Although tomotherapy needs long treatment times, the recent Hi‐Art system includes an additional shielding composed of lead disks and of a 20 cm thick tungsten system. These components enable to reduce peripheral doses delivered far from the target in comparison with conventional accelerators. As former tomotherapy systems did not include this shielding, they used to deliver higher peripheral doses.^20^ This shielding is the reason why tomotherapy better spares tissues located far from PTV in our work. Ramsey *et al*.^21^ show that even if tomotherapy irradiation time is 5–15 times the one of IMRT, it delivers lower or equivalent dose levels outside the treatment edges. The use of FFF beams in tomotherapy also decreases stray radiation coming from the accelerator head. Recently, D’agostino *et al*.^22^ show that, for a prostate cancer, tomotherapy delivers similar healthy organ doses to those of a 10 MV IMRT treatment taking into account the photon contribution to the dose. Those authors also show, for a head and neck treatment, that tomotherapy and 6 MV VMAT equally expose normal tissues without considering on‐board imaging doses. This result is different from the larger VMAT doses obtained in this work compared to tomotherapy; however, as the design of the tomotherapy unit is not described in,^21^ the unit may have a less effective shielding than recent designs as used in the present work. Furthermore, on‐board images in tomotherapy performed with MVCT which exposes the pelvic organs to an additional dose of 10 mGy according to these authors were considered in.^22^ Kowalik *et al*.^23^ also indicate that tomotherapy better spares every organ in comparison with IMRT for a prostate treatment. Finally, Jeraj *et al*.^24^ and Balog *et al*.^25^ report that the tomotherapy shielding considerably reduces the radiation level inside the treatment room and the stray radiation delivered to the patient. Thus, the leakage radiation in the patient plan comes in majority from leakage through MLC closed leaves and jaws. This radiation reaches the patient close to the beam edges; this can explain the higher dose levels obtained in our study using tomotherapy in comparison with VMAT. This has mostly been observed for the 5‐year‐old phantom as the treatment has been performed with fixed jaws triggering a larger penumbra in the craniocaudal direction than with dynamic jaws.^26^, ^27^ This result is illustrated by Fig. 9 showing calculated profiles on the 5‐year‐old phantom using fixed and dynamic jaws with the Tomotherapy TPS. In our study, this TPS proves to be reliable for the out‐of‐field dose calculation: it allows to confirm the increase in penumbra triggered by static jaws and the slight increase of peripheral dose. The dose difference between the two profiles decreases with distance to the target reaching 7% at 30 cm from field edge.

As a consequence, our study reports an increase in dose to the pelvis: this result raises concern given the gonads radio‐sensitivity that may lead to an increased side effects risk. Moreover, a larger dose to the heart has been measured in tomotherapy in comparison to VMAT and 3DCRT for this phantom. This increase in dose can be involved in a higher cardiac risk following treatment.^28^ Finally, the dynamic jaw mode enables to reduce the dose in beam periphery^26^; this is observed up to approximatively 17.5 cm from field edge in our work.

Moreover, in this study, higher doses are observed in the caudal direction close to the target with tomotherapy and for the two phantoms, that is, for the two jaw modes. For instance, for the treatment of the 10‐year‐old phantom, the abdominal‐pelvic organs located near PTV (uterus, ovaries, digestive system, and rectum) received higher doses compared to those measured in VMAT. Thus, tomotherapy unit’s leakage seems to contribute mainly in this direction. This leakage is more important in proximity of the PTV because when the pelvic area is considered in whole, the VMAT mean doses are higher than those delivered in tomotherapy.

Finally, 3DCRT treatments have been performed using 6 MV beams, which represent the worst‐case scenario in terms of photon leakage dose. This is due to the higher MU needed to deliver the dose with this energy.^7^ Despite this observation, it has been shown in this study that this technique better spares the normal tissue located far from PTV than modern techniques.

In our study, only doses delivered by treatment beams were evaluated. However, important dose levels can be delivered by daily on‐board tomotherapy MVCT using 3.5 MV beams.^22^ In fact, Shah *et al*.^29^ show that doses exceeding 1 cGy per fraction are delivered to the bladder and the rectum during a prostate treatment and Nagata *et al*.^30^ report that, for some organs, this system delivers doses up to 1.9 cGy per fraction to parotid for a head and neck treatment. Finally, De Saint‐Hubert *et al*.^28^ highlight that tomotherapy MVCT doses can reach up to 2 cGy per fraction to the thyroid, which is almost four times the doses delivered by a kV‐CBCT imager. Thus, the conclusion drawn from our study regarding tomotherapy exposure of normal tissues in regards of other techniques could be different while taking into account on‐board imaging doses.

### 4.B Performance of TPS algorithms

Regarding the Eclipse™ TPS, the Acuros^®^ algorithm is found to calculate doses on a larger volume than AAA for both VMAT and 3DCRT, this result was also reported by Mille *et al*.^31^ Their study concludes that this TPS is not suitable for distant organ dose determination as well. Likewise, large discrepancies in DVH were also found in the present study. As DVH is one of the main clinical tools used to assess treatment plan quality, the user has to keep in mind the limitation of TPS calculation for organs partially located in treatment beams.

Besides, the dose decrease away from PTV is not correctly calculated by the two algorithms of this TPS leading to large peripheral dose underestimation compared to measurements. This observation is in agreement with results of the literature.^7^, ^32^, ^33^, ^34^ Specifically, underestimations above 40% have also been reported by Howell *et al*.^35^ with AAA between 3 cm and 11 cm from field edge in 3DCRT and mean discrepancies of 14% have been shown by Taddei *et al*.^36^ with AAA in 3DCRT between 1 cm and 8 cm from beams. Those discrepancies are superior to those shown in our work in 3DCRT where a difference of −1.46% was obtained between 1 cm and 8 cm from field edge. In accordance with our results, Mille *et al*.^31^ also have a satisfying agreement between TPS calculations, measurements, and Monte Carlo simulations up to 5–8 cm from field edge in the case of a fixed squared beam treatment.

Finally, in our work, a different behavior of AAA was observed depending on radiotherapy technique and accelerator. As AAA does not model head leakage^37^ which increases with beam‐on time and depends on accelerator head design; this can explain the large peripheral dose underestimation obtained with VMAT using the Clinac 2100CS (Fig. 5). The work of Wang and Ding^38^ also reports large underestimation of the AAA algorithm (40–80%) in comparison with MC calculation for organs located far from PTV with IMRT plans (doses inferior to 0.5% of prescribed dose). The larger discrepancies are observed at 2 cm in depth. But contrary to our observations, they report a good agreement between AAA and MC up to 15 cm from field edge for VMAT plans. In a second study,^39^ these authors report a consistent underestimation of AAA of 30–50% for VMAT plans (doses less than 1% of the prescribed dose). They estimated that the Eclipse calculations need to be multiplied by a factor 2 to better represent the doses delivered to normal tissues.

At the contrary, the Tomotherapy TPS calculates doses on the whole CT volume in our study and agreement with measurement is good on this entire volume (mean difference less than 11% except for the head and neck region). A minor dose underestimation of the TPS is observed for healthy organs. Those results confirm that this TPS is well adapted to calculate doses to remote organs up to 30 cm from beam axis. Similar results have been reported by Schneider *et al*.^40^: they show that Tomotherapy TPS provides calculated doses in agreement with measurements (with a 50% tolerance) up to 35 cm from axis for a prostate treatment. Beyond this distance, this TPS tends to underestimate delivered doses. They conclude that in order to evaluate the risks associated with normal tissue exposure, the Tomotherapy TPS can be used up to 35 cm from axis. The precision of this TPS away from beam edge is induced by the use of point kernels calculated in a large volume around interaction point. In fact, the works of Mackie *et al*.^41^ and Papanikolaou *et al*.^42^, on which this convolution/superposition algorithm is based, report point kernels calculated up to 30 cm from the voxel center in the lateral direction and up to 85 cm in depth. The lateral distance is in agreement with our results and those obtained by Schneider *et al*.^40^

## 5 Conclusion

In this work, out‐of‐field doses delivered by four radiotherapy techniques have been evaluated by means of film measurements in pediatric anthropomorphic phantoms. Modern techniques enable higher dose conformation compared to 3DCRT; however, this improvement is done at the cost of higher peripheral doses. This study points out a factor of 3 on dose between modern treatments and 3DCRT for organs located far from PTV. This larger exposure raises major concern as it might increase the risks of developing adverse effects following radiotherapy especially for pediatric patients surviving long after the treatments. Among advanced radiotherapy techniques, the latest generation Varian Halcyon system seems a promising treatment option as delivering lower dose levels than conventional accelerator and incorporating kV‐CBCT imager. This technique was also the most efficient in terms of treatment delivery time. To our knowledge, our study is the first to report healthy tissue doses delivered with the new Varian Halcyon system. It enables to situate this new treatment option in relation to the other older techniques. The original methods developed and applied to renal pediatric treatments in this work can be used to study other radiotherapy techniques or tumor localization. The conclusions obtained in this work cannot be easily extended to other localizations in particular regarding the dose distribution close to the PTV as it depends on PTV size and its relative distance to normal organs. However, the results obtained away from PTV are more general as the doses delivered are highly dependent on accelerator head design and treatment efficacy and less on morphology. These general findings are important to provide clinical data regarding modern pediatric radiotherapy treatments using latest generation accelerators.

In light of the results presented in this study, it would be interesting to complete the dosimetric comparison between techniques by adding daily imaging dose determination as they largely contribute to increase the exposure of healthy tissues to radiation.

Finally, TPS performances were evaluated in terms of normal tissue dose calculations. Unlike Eclipse™(AAA and Acuros^®^), the Tomotherapy TPS enables a precise dose calculation up to 30 cm from field edge. This study is useful in providing clinical information on the uncertainties of healthy organ doses calculated by two modern treatment planning system for which very few data are available in literature.
